# Li-ion transport in two-dimensional nanofluidic membranes

**DOI:** 10.1186/s40580-024-00465-y

**Published:** 2024-12-12

**Authors:** Gyu Won Kim, Minwoo Lee, Jihong Bae, Jihoon Han, Seokmin Park, Wooyoung Shim

**Affiliations:** 1 Department of Materials Science and Engineering, Yonsei University, Seoul, 120-749 Korea; 2 Center for Multi-Dimensional Materials, Yonsei University, Seoul, 03722 Korea; 3 Center for Nanomedicine, Institute for Basic Science (IBS), Seoul, 03722 Korea; 4 Graduate Program of Nano Biomedical Engineering (NanoBME), Advanced Science Institute, Yonsei University, Seoul, 03722 Korea

**Keywords:** Lithium resources, Two-dimensional membranes, Nanofluidics, Ion channel, Ion selectivity

## Abstract

The growing demand for lithium, driven by its critical role in lithium-ion batteries (LIBs) and other applications, has intensified the need for efficient extraction methods from aqua-based resources such as seawater. Among various approaches, 2D channel membranes have emerged as promising candidates due to their tunable ion selectivity and scalability. While significant progress has been made in achieving high Li^+^/Mg^2+^ selectivity, enhancing Li^+^ ion selectivity over Na^+^ ion, the dominant monovalent cation in seawater, remains a challenge due to their similar properties. This review provides a comprehensive analysis of the fundamental mechanisms underlying Li^+^ selectivity in 2D channel membranes, focusing on the dehydration and diffusion processes that dictate ion transport. Inspired by the principles of biological ion channels, we identify key factors—channel size, surface charge, and binding sites—that influence energy barriers and shape the interplay between dehydration and diffusion. We highlight recent progress in leveraging these factors to enhance Li^+^/Na^+^ selectivity and address the challenges posed by counteracting effects in ion transport. While substantial advancements have been made, the lack of comprehensive principles guiding the interplay of these variables across permeation steps represents a key obstacle to optimizing Li^+^/Na^+^ selectivity. Nonetheless, with their inherent chemical stability and fabrication scalability, 2D channel membranes offer significant potential for lithium extraction if these challenges can be addressed. This review provides insights into the current state of 2D channel membrane technologies and outlines future directions for achieving enhanced Li^+^ ion selectivity, particularly in seawater applications.

**Graphical Abstract:**

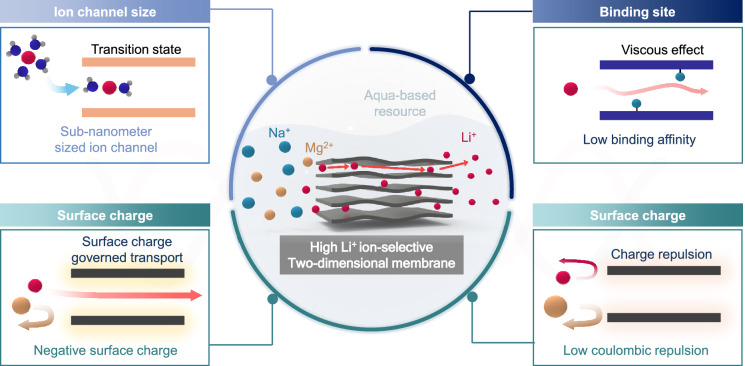

## Introduction

Lithium is a crucial element in modern society and is used across various fields, such as lithium-ion batteries (LIB), ceramics, and glass manufacturing [1]. Particularly, LIB holds approximately 40% of the global battery market due to their high specific energy density (100–265 Wh/kg), specific power (250–340 W/kg), and excellent cycle performance (400–1200 cycles), making lithium a critical resource [2–4]. Lithium-ion batteries are primarily utilized in electric vehicles and portable electronic devices. With advancing technologies and increasing energy consumption, global lithium demand is projected to grow steadily, rising from 165 kt in 2023 to 1326 kt by 2040—a nearly eightfold increase [5–9]. Therefore, ensuring a sustainable lithium supply will become an increasingly significant challenge.

The major global lithium resources are found in hard-rock ores and aqua-based resources such as brine and seawater, where lithium exists in the form of dissolved salts. While hard-rock ores have been more widely exploited due to their higher lithium concentration (1–4 wt%), there may be supply limitations [3, 10, 11]. In contrast, aqua-based resources, including seawater (~ 230 Gt of lithium), contain far more abundant lithium reserves than hard-rock ores [1, 12]. As global demand for lithium continues to rise, extracting Li^+^ ions from aqua-based resources will likely be essential to meet future needs.

The primary technology for extracting lithium from aqua-based resources has traditionally been evaporation technology. This method involves three major steps: i) Li^+^ ion enrichment via solar evaporation, ii) purification to remove non-Li^+^ ions, and iii) precipitation to crystallize Li^+^ ions into Li_2_CO_3_ [13, 14]. However, this process is constrained by the low concentration of Li^+^ ions in aqua-based resources (0.1–0.2 ppm for seawater, 200–700 ppm for brine) [4, 15], requiring approximately 100–800 m^3^ of water to produce 1 ton of lithium carbonate. As a result, large evaporation ponds are needed, and Li^+^ ion enrichment takes 10–24 months to complete [1, 5, 6]. Additionally, chemical agents used during the purification process to achieve high-purity Li_2_CO_3_ pose potential environmental risks [16, 17]. These inefficiencies and sustainability concerns highlight the need for new methods of Li^+^ ion extraction from aqua-based resources.

In response to the growing demand for efficient Li^+^ ion extraction from aqua-based sources, several methods have been proposed, including adsorption [18, 19], pulsed electrochemical intercalation [20], liquid extraction [21], and ion sieving membranes [22, 23]. Among these, ion sieving membranes and liquid extraction exhibit promising efficiency due to their single-step, continuous operation, while adsorption and pulsed electrochemical intercalation are hindered by their two-step processes. Furthermore, the lower reliance on chemical reagents observed in ion sieving membranes and pulsed electrochemical intercalation suggests their potential for reduced environmental impact compared to other reagent-intensive methods [1, 19, 24–26]. As a result, ion-sieving membranes have attracted significant attention due to their high energy efficiency, and minimal use of chemical agents, making them a promising alternative to conventional enrichment and purification methods [27, 28].

Ion sieving membranes can be categorized into two main types based on their ion transport mechanisms: ion exchange membranes and nanofluidic membranes. Ion exchange membranes feature channel sizes comparable to the size of ion species, resulting in high selectivity ($S=\frac{{P}_{i}}{{C}_{1i}}/\frac{{P}_{k}}{{C}_{1k}}$, where $S$ is selectivity of ion $i$ over ion $k$, ${P}_{i}$ is permeability of ion $i$, ${C}_{1i}$ is initial concentration of ion $i$). However, due to their high resistance, ion transport, or permeability (${P}_{i}=\frac{({C}_{1}-{C}_{0})\cdot V}{A\cdot t}$, where ${P}_{i}$ is permeability of ion $i$, ${C}_{1}$ is permeated concentration of ion $i$, ${C}_{0}$ is initial concentration of ion $i$) [29], is often very low, making them inefficient for Li^+^ ion extraction. In contrast, nanofluidic membranes, with their relatively larger channel sizes, offer higher permeability and are better suited for Li^+^ ion extraction [30]. This review aims to explore Li^+^ ion-selective nanofluidic membranes, classifying them based on channel dimensions. We will also outline the challenges and future directions for enhancing Li^+^ ion selectivity in 2D channel membranes from both material and performance perspectives.

## Properties of Li^+^ ion selective nanofluidics membranes with channel dimensions

**Fig. 1:**
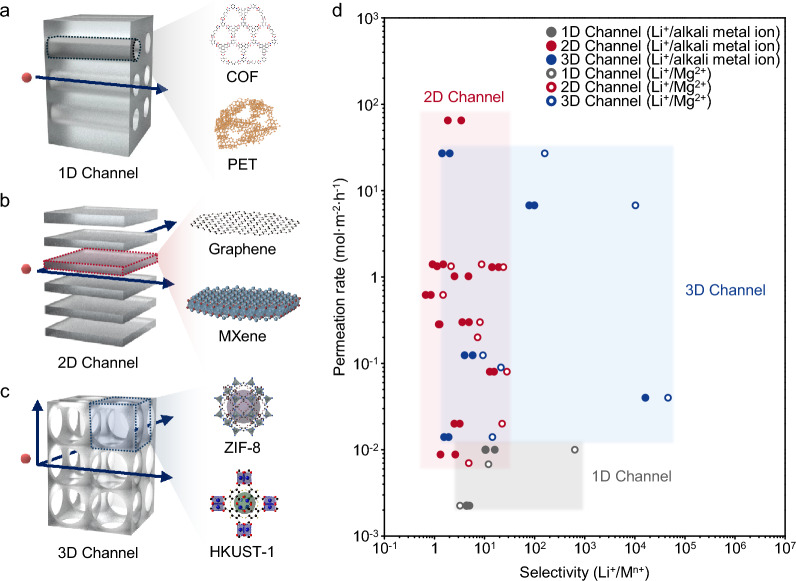
Recent studies of Li^+^ ion selective nanofluidics membrane. **a** Schematic of 1D channel membrane (left) and examples of materials for 1D channel membrane. COF, Covalent-organic framework; PET, Polyethylene tetraphthalate. **b** Schematic of 2D channel membrane (left) and examples of materials for 2D channel membrane. **c** Schematic of 3D channel membrane (left) and examples of materials for 3D channel membrane. **d** Performance of Li^+^ ion selective membrane with different channel dimensions. References and values for all plotted data in the graph are in

Extensive research has been conducted to implement high Li^+^ ion selectivity by tailoring the ion channel dimensions of nanofluidic membranes. Nanofluidic membranes can be classified based on ion transport direction into one-dimensional (1D), two-dimensional (2D), and three-dimensional (3D) channel membranes (Fig. 1a-c) [31, 32]. In 1D channel membranes, ion transport is restricted to a single direction. Notable examples include aligned Covalent-Organic Framework (COF) nanosheets arranged in a columnar fashion [33] and polyethylene terephthalate (PET) membranes produced by ion track-etching (Fig. 1a) [34]. While 1D channel membranes allow for predictable ion-transport direction, their practical use is limited by high production costs and the challenges of scaling up [35].

3D channel membranes allow ion transport in three directions and are typically composed of porous structures with interconnected pores, such as Metal–Organic Frameworks (MOFs) [36] and Physical-Organic Frameworks (POFs) (Fig. 1c) [17]. These materials feature nano- or angstrom-sized pores, which provide high selectivity. MOFs, in particular, offer the advantage of tunable geometric structures and pore sizes based on the choice of metal ions and ligands [37]. However, the high pore density of these membranes often results in low mechanical strength [38], and their stability in aqueous environments is limited [27, 39, 40], which poses a challenge for their use in Li^+^ ion extraction from aqua-based resources.

2D channel membranes, where ion transport occurs along two axes, are typically composed of laminar membranes formed from stacked atomic-scale monolayers such as graphene derivatives, MXenes, and clay minerals (e.g., vermiculite, montmorillonite) (Fig. 1b). Due to the intrinsic properties of these materials, 2D channel membranes can offer relatively uniform and homogeneous channel sizes, making them potentially highly selective. Furthermore, compared to 1D and 3D channel membranes, 2D channel membranes are easier to fabricate and scale up [41–43]. Additionally, 2D channel membranes can be tuned for optimized Li^+^ ion extraction via various methods, including molecule implantation, channel size control, and surface modification [44].

2D channel membranes also show promise not only from a materials perspective but also in terms of membrane performance, such as selectivity and permeability. Figure 1d presents a graph of Li^+^ ion selectivity and permeability across nanofluidic membranes with different channel dimensions, based on studies published in the past decade. Li^+^ ion selectivity is shown relative to other cations present in aqua-based resources (Table 1), specifically Mg^2+^ ions prevalent in brine and alkali metal ions (Na^+^, K^+^) found in seawater. 1D channel membranes generally exhibit lower selectivity and permeability, indicating limitations in practical Li^+^ ion extraction. In contrast, 2D and 3D channel membranes demonstrate higher permeability and selectivity, indicating their potential for use in Li^+^ ion extraction technologies Table 2.

**Table 1:** Ion composition in aqua-based resources (g/L)

Water resource		Li^+^	Mg^2+^	Na^+^	K^+^	Ca^2+^	Ref
Brine	Olaroz Salar Brine, Argentina	1.01	**2.00**	98.85	6.22	0.51	[88]
	Uyuni Salar Brine, Bolivia	0.84	**16.7**	105.4	15.7	3.33	[89]
	Atacama Salar Brine, Chile	3.02	**17.6**	61.9	28.2	0.41	[90]
	Qarhan Salt Lake, China	0.279	**108.5**	2.281	0.76	0.107	[91]
	Yiliping Salt Lake, China	0.33	**26.29**	78.02	15.11	–	[92]
	Longmucuo Salt Lake, China	0.865	**75.41**	11.10	15.59	0.12	[93]
	West Taijinar Salt Lake, China	0.784	**60.58**	21.01	16.00	0.185	[94]
	East Taijinar Salt Lake, China	6.75	**85.47**	10.42	7.69	–	[95]
Seawater	Normal Seawater	1.83E-4	1.28	**10.8**	0.40	0.41	[96]
	Mediterranean sea	–	1.30	**18.0**	0.04	0.04	[97]
	Arabian Gulf, Kuwait	–	1.77	**15.9**	0.46	0.50	[97]
	Red Sea	1.8E-4	1.61	**10.57**	0.67	0.48	[98]

**Table 2:** References of Li^+^ ion selective nanofluidics membranes

Channel dimension & Membranes		Li^+^ permeation rate (mol·m^−2^·h^−1^)	Li^+^/Mg^2+^ selectivity	Li^+^/Na^+^ selectivity	Li^+^/K^+^ selectivity	Ref
1D channel membrane	COF-4EO	0.0068	12	–	–	[33]
	PET	0.03	633.98	10.46	16.02	[34]
	NP5-functionalized PET	0.0025	3.24	4.4	5	[99]
2D channel membrane	MXene(Ti_3_C_2_T_*x*_)-PSS	0.08	28	15.5	12.7	[82]
	GO-Tannic acid	0.62	1.49	0.85	0.67	[22]
	GO-PSS	65	–	1.85^[a]^	3.43^[a]^	[39]
	MXene(Ti_3_C_2_T_*x*_)-PSS	0.02	–	2.5	3.2	[100]
	pGO/nMXene	1.02	8.07	2.52	4.78	[101]
	Vermiculite	0.3	8.1	3.6	4.9	[73]
	Vermiculite-SPVA	1.3	23.8	14.1	19.1	[73]
	MXene(Ti_3_C_2_T_*x*_)	1.4	8.75	0.92	1.5	[102]
	GO	1.33	2.15	1.13	–	[102]
	LDH(ZnAl-NO_3_)	0.007	4.85	–	–	[67]
	MXene(Ti_3_C_2_T_*x*_)	0.2	7.29^[a]^	–	–	[29]
	MXene(Ti_3_C_2_T_*x*_)-EDTA-1.5	0.02	22.44^[a]^	–	–	[29]
	MXene(Ti_3_C_2_T_*x*_)	0.283	–	1.27	1.22	[75]
	Thermal treated MXene(Ti_3_C_2_T_*x*_) at 180 °C	0.0088	–	1.32	2.61	[75]
3D channel membrane	10% Crown-POF based mixed matrix membrane	0.014	14.39	1.56	1.92	[17]
	LLTO(Li_0.33_La_0.56_TiO_3_)	0.04	45916^[a]^	16277^[a]^	–	[103]
	Li_1.5_Al_0.5_Ge_1.5_(PO_3_)_4_/PEDOT:PSS + MWCNTs-NH2	0.124	9.3	4	5.8	[104]
	COF-PA	0.0897	21.3^[a]^	–	–	[66]
	MOF(UIO-67)	27.01	159.4	2.02	1.42	[60]
	MOF(HKUST-1)-PSS	6.75	10,296	78	99	[36]

[a] mixed salt solution selectivity

Given the lower performance and scalability challenges of 1D channel membranes, as well as the instability and mechanical strength issues faced by 3D channel membranes in aqueous environments, 2D channel membranes are considered the most promising for practical Li^+^ ion extraction. In this context, we will explore the factors affecting Li^+^ ion extraction rates in 2D channel membranes and compare Li^+^ ion extraction efficiencies relative to Na^+^ and Mg^2+^ in brine and seawater.

## Principles of Li^+^ ion selectivity through the membrane

The selective transport of specific ions is often related to their mobility. The mobility (*μ*) of ions in bulk aqueous solutions is influenced by factors such as the viscosity (*η*) of the solution, the radius of the hydration sphere (*a*), and the valence of the ion (*z*), and can be expressed by the following equation [45]:

$$\mu =\frac{ze}{6\pi \eta a}$$

For ions with the same valence, the radius of the hydration sphere and hydration energy are typically inversely proportional to the bare ion size. In this regard, the Li^+^ ion, which has the smallest bare ion size (0.6 Å) among alkali metal ions, has a relatively large hydration sphere radius (3.82 Å) [46]. Additionally, according to the equation above, the relatively low valence of Li^+^ ion leads to lower ion mobility compared to alkaline earth metal ions with higher valences. Therefore, Li^+^ ions generally exhibit lower mobility in bulk solution than Na^+^ and Mg^2+^ ions.

In contrast to ion transport in bulk aqueous solutions, membrane channels act as selective barriers, preferentially allowing certain ions to pass [44, 47]. While ion mobility in micrometer-scale channels may resemble that in bulk aqueous solution [48], in nanometer- and subnanometer-scale channels, ion mobility is affected by complex interactions between the channel walls and the ions [49, 50]. Therefore, membranes with nanoscale and subnanoscale channels—referred to as nanofluidic membranes—are considered a crucial factor in achieving Li^+^ ion selectivity. Understanding the ion transport mechanisms in nanofluidic membranes is essential for realizing high Li^+^ ion selectivity.

**Fig. 2:**
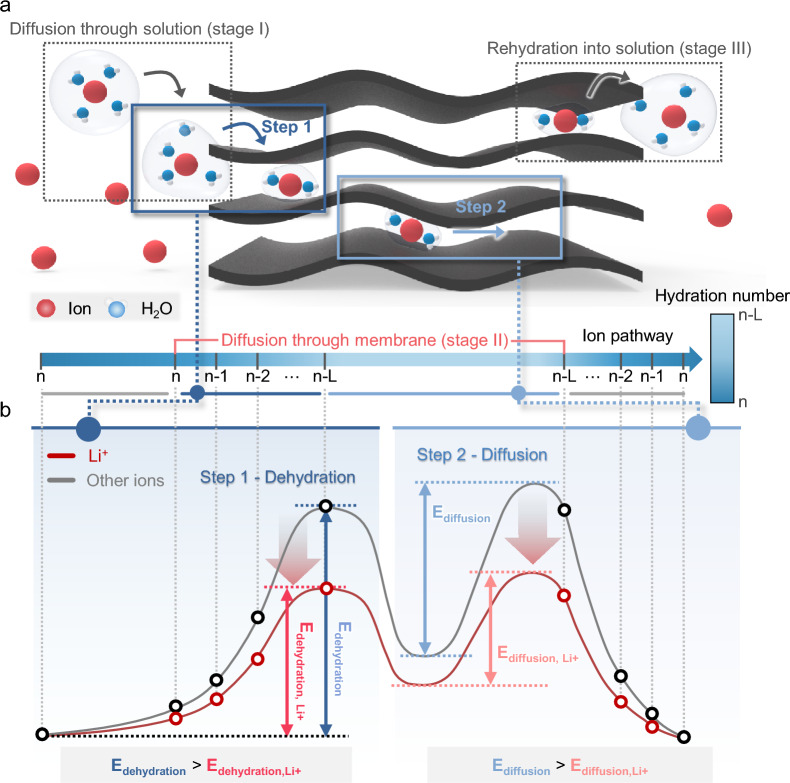
Principle of Li^+^ ion selectivity through the membrane. **a** Schematic of ion permeation process through 2D channel membrane. The ion permeation process can be divided into diffusion through the solution and diffusion through the membrane; the ion permeation process can be divided into diffusion through the solution, diffusion through the membrane, and hydration into the solution. In practice, diffusion through the membrane can be a rate-limiting step that is divided into i) the dehydration step and ii) the diffusion step. The hydration number of ions can be changed in the ion permeation process. **b** Schematic of the energy barrier for two steps in diffusion through the membrane. Each ion can experience an individual energy barrier based on interaction with the membrane. Li^+^ ion selectivity can be enhanced when the energy barrier for each step is reduced only for Li^+^ ion

The process of ion transport through nanofluidic membranes is generally divided into three stages: diffusion through solution (Stage I), diffusion through the membrane (Stage II), and rehydration into solution (Stage III) [51–53]. Each stage is influenced by the ion’s hydration interactions. In Stage I, fully hydrated ions (with a hydration number of *n*) move through the solution; in Stage II, partial dehydration and the transport of partially dehydrated ions (hydration number from *n* to *n–L*) occur; and in Stage III, the partially dehydrated ions rehydrate (hydration number from *n–L* to *n*). The rehydration step in Stage III, being the reverse of Stage II, is unlikely to have a significant effect on ion selectivity. Moreover, since the diffusion coefficient in solution (Stage I) is several orders of magnitude larger than the diffusion coefficient within the membrane (Stage II and III), the ion transport through the membrane (Stage II) is the rate-limiting step in the overall process (Fig. 2a) [54].

The diffusion through the membrane (Stage II) can be further divided into two steps: the dehydration step, where hydrated ions lose part of their hydration shell while entering the membrane (change in hydration number from *n* to *n–L*), and the diffusion of the partially dehydrated ions through the membrane (hydration number *n–L*). Ion selectivity arising from this process can be explained using the Solution-Diffusion Model and Eyring’s Transition-State Theory (TST).

According to the Solution-Diffusion Model, permeability (*P*) is expressed as the product of the partition coefficient (*K*), representing the process of the molecule entering the membrane, and the diffusion coefficient (*D*), representing the diffusion process within the membrane [55, 56]:

$$P=K\cdot D$$

The Solution-Diffusion Model provides a framework for understanding the two-step process of solute permeation through membranes. However, it has limitations in explaining molecular-level phenomena during ion permeation (e.g., sorption interactions and partial dehydration of ions). As such, the model struggles to account for the selectivity between similarly sized and charged ions, such as mono-/monovalent or mono-/divalent cations [51, 53, 57].

Eyring’s Transition-State Theory describes ion transport through membranes as a series of activation energy barriers [54]. This theory quantifies ion dehydration in terms of a rate constant, which plays a critical role when ions traverse sub-nanoscale nanofluidic channels. Furthermore, understanding the interaction between the membrane and ions in terms of activation energy offers a way to explain selectivity between mono-/monovalent and mono-/divalent cations, which the Solution-Diffusion Model cannot adequately address [51, 53].

Based on these two models, ion diffusion through membranes can be understood as passing through two consecutive energy barriers: i) dehydration and ii) diffusion. Thus, Li^+^ ion selectivity can be attributed to differences in the magnitude of the energy barriers at each step. By selectively lowering the energy barrier for Li^+^ ions, there is potential to enhance Li^+^ ion selectivity (Fig. 2b).

The energy barriers associated with ion transport through membranes are influenced by factors such as ion dehydration, electrostatic interactions between the ion and the membrane channel, short-range steric interactions, and long-range van der Waals interactions [30]. Ion dehydration, which is closely related to the intrinsic properties of ion species (i.e., hydration energy), can significantly affect ion mobility. The impact of dehydration becomes especially pronounced when ions have different charges. For example, the hydration energy of alkali metal ions such as Li^+^ and Na^+^ ions range from –250 to –475 kJ/mol, whereas for alkaline earth metal ions like Mg^2+^ ion, the range is significantly higher, from –1250 to –2395 kJ/mol [58]. Therefore, Li^+^/Mg^2+^ selectivity may largely stem from the effects of dehydration and differences in hydration energy.

In contrast, Li^+^ and Na^+^—both alkali metal ions—have similar hydration energies and hydrated radius caused by the same ionic charges and similar bare ion sizes, making it more difficult to achieve Li^+^ ion selectivity over Na^+^ ion compared to Mg^2+^ ion. In this regard, understanding the origins of Li^+^/Na^+^ selectivity requires consideration of various interactions which is largely dependent on the properties of membrane channel wall [53, 59]. By analyzing the property differences between alkali metal and alkaline earth metal ions, we can gain insight into the mechanisms underlying Li^+^/Mg^2+^ and Li^+^/Na^+^ selectivity during membrane permeation.

## Origin of Li^+^/Mg^2+^ selectivity

The hydration interaction can be often proportional to valence of ion and inversely proportional to bare ion radius. The bare ion radius of Mg^2+^ ion is 0.65 Å, which is only slightly larger than that of Li^+^ ion (0.6 Å). Despite of similar bare ion size, as a divalent cation, Mg^2+^ ion carries a higher charge number than Li^+^ ion, resulting in a significantly higher hydration energy (Li^+^: –475 kJ/mol, Mg^2+^: –1830 kJ/mol) and a larger hydrated ion size (Li^+^: 7.64 Å, Mg^2+^: 8.56 Å) [46, 58]. The differences in hydration energy and ion valence can lead to distinct interactions between the channel walls and Li^+^ or Mg^2+^ ions. These interactions manifest differently during the dehydration and diffusion steps, contributing to Li^+^/Mg^2+^ selectivity.

The large hydration energy of Mg^2+^ ion plays a critical role in Li^+^/Mg^2+^ selectivity during the dehydration step. If the membrane channel size is smaller than the hydrated ion size of Mg^2+^ ion, only Li^+^ ion can selectively pass through the membrane. Additionally, the negative surface charge of the membrane assists in stripping the hydration shell from ions. While it is difficult for Mg^2+^ ion to shed its hydration shell due to its large hydration energy, Li^+^ ion, with its relatively lower hydration energy, can selectively lose its hydration shell and enter the membrane in a partially dehydrated state, thereby enhancing Li^+^/Mg^2+^ selectivity [60].

Another method to improve Li^+^ ion selectivity involves using the surface charge of the membrane to reject Mg^2+^ ions. Because Mg^2+^ ion has a higher ion valence, it experiences stronger repulsive interactions when the membrane surface has a positive charge. This repulsive interaction can be an effective means to enhance Li^+^/Mg^2+^ selectivity. However, careful tuning is required to avoid hindering Li^+^ ion permeation as well.

The higher ion valence of Mg^2+^ ion also plays a significant role in Li^+^/Mg^2+^ selectivity during the diffusion process. Negative binding sites within the membrane channels can bind Mg^2+^ ion more strongly than Li^+^ ion, slowing down Mg^2+^ ion diffusion relative to Li^+^ ion. By leveraging these strategies, Li^+^/Mg^2+^ selectivity can be greatly improved. This approach, which carefully considers ion valence, hydration energy, and interactions with the membrane, is key to achieving high Li^+^ ion selectivity.

### Li^+^/Mg^2+^ selectivity in dehydration step with 2D channel membrane

This section explores four scenarios: (i) improving Li^+^ ion selectivity solely through channel size without surface charge effects, (ii) enhancing Li^+^ ion selectivity by reducing channel size with a negatively charged surface, (iii) using a positively charged surface to repel divalent ions and improve Li^+^ ion selectivity, and (iv) enhancing Li^+^ ion selectivity by incorporating functional groups or molecules that bind preferentially to Li^+^ ion within the channel.

First, when entering channels smaller than the hydrated ion size, Mg^2+^ ion requires more energy to remove its strongly bound hydration shell compared to Li^+^ ion. As a result, simply reducing the channel size without adjusting the surface charge can lead to Li^+^/Mg^2+^ selectivity.

**Fig. 3:**
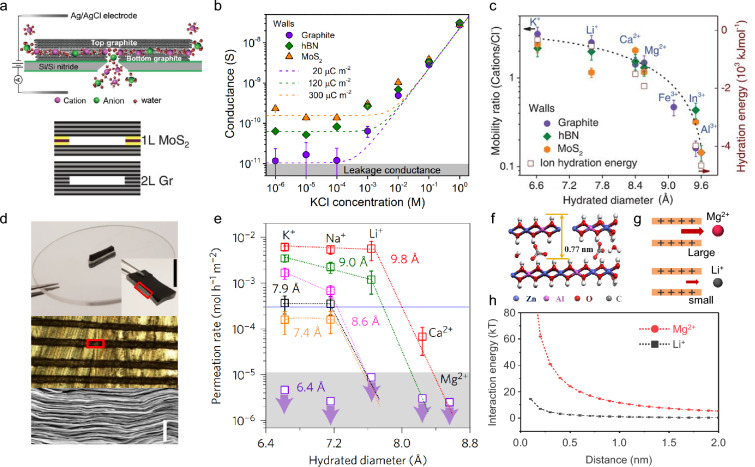
Li^+^/Mg^2+^ selectivity in the dehydration step with 2D channel membrane. **a** Schematic of fabricated angstrom-sized slit channel. The size of the channel is different from the spacer material. 1L MoS_2_, ~ 6.6 Å; 2L graphene, 6.7 Å. **b** Conductance at various concentrations with various channel wall materials. For KCl concentrations ≥ 10^−1^ M, the surface charge of the channel can be neglected. **c** Mobility ratio(cation/Cl^−^) of hydrated cations under the condition of neglected surface charge. **d** Picture of graphene oxide membrane embedded in epoxy resin (top). Optical micrograph of the cross-section of physically confined graphene oxide (PCGO) membrane (middle). Scanning electron microscopy image of the red squared region (bottom). **e** Permeation rates through the PCGO membrane of hydrated cations with different channel sizes. **f** Schematic of cross-section layered double hydroxide (LDH) membrane. **g** Schematic of electrostatic repulsion caused by LDH membrane against Li^+^ ion and Mg^2+^ ion. **h** Surface element integration model predictions of electrostatic repulsive force of charged LDH membrane surface against Li^+^ ion and Mg^2+^ ions. a-c) Reproduced with permission [48]. Copyright 2017, The American Association for the Advancement of Science. **d**, **e** Reproduced with permission [62]. Copyright 2017, Springer Nature Limited. **f**–**h** Reproduced with permission [67]. Copyright 2023, American Institute of Chemical Engineers

For example, Esfandiar et al. observed this phenomenon by constructing flat, angstrom-sized slits that enhanced ion selectivity without altering surface charge [48]. They used double-layer graphene and monolayer MoS_2_ as spacers between graphene, hBN, and MoS_2_ crystals to fabricate slit channels approximately 6.6–6.7 Å in size (Fig. 3a). To focus on the effect of channel size on ion selectivity, surface charge effects were excluded. At high solution concentrations, surface charge effects are screened by counter-ions within the channel [61]. The authors measured conductance and zero-current potential in a concentration range where surface charge effects were screened, representing bulk-like behavior (Fig. 3b). The measured zero-current potential was converted into the cation/Cl^–^ mobility ratio using the Henderson equation. The results showed that ions with larger hydration energies exhibited lower cation/Cl^–^ mobility ratios within the fabricated channel size range (6.6–6.7 Å) (Fig. 3c). From this, they concluded that even in the absence of surface charge, Li^+^/Mg^2+^ selectivity could be achieved by controlling channel size.

Li^+^/Mg^2+^ selectivity through reduced channel size can also be observed in membranes with a negatively charged surface. Graphene oxide (GO) membranes, for example, feature functional groups such as hydroxyl, carboxyl, and epoxy groups on their surfaces, which deprotonate in water to form negatively charged surfaces. Abraham et al. tested ion permeation through GO membranes with controlled interlayer spacing [62]. To prevent further swelling in water, the adjusted GO membranes were physically confined using epoxy (Fig. 3d). By varying the hydration level through humidity control, the interlayer spacing of GO was tuned. The actual channel sizes, calculated by subtracting the graphene monolayer thickness (3.4 Å) from the measured interlayer spacing, ranged from 6.4 to 3 Å. Permeation tests revealed that Li^+^ ions permeated several orders of magnitude faster than Mg^2+^ ions when the channel size was between 6.4 Å and 5.6 Å (Fig. 3e). However, for smaller channel sizes, detectable Li^+^ ion transport ceased, likely due to the high hydration energy of Li^+^ ion, which prevents permeation through excessively small channels. Thus, careful control of channel size is essential.

In many salt-rejecting membranes, positive surface charges are introduced via coatings or functional groups to repel cations [63, 64]. This strategy can also be applied to improve Li^+^/Mg^2+^ selectivity [65, 66]. Li et al. successfully fabricated micro-sized nitrate ZnAl Layered Double Hydroxide (LDH) membranes [67]. LDH is a 2D material with positively charged laminates and intercalated anions (Fig. 3f). The authors confirmed that exfoliated LDH nanosheets have positively charged surfaces through zeta potential measurements. These positively charged surfaces exert repulsive forces on cations via electrostatic double-layer interactions. The surface element integration model calculated the electrostatic repulsive interaction energy between the positively charged surface and cations. The results showed that divalent cations, such as Mg^2+^ ion, experience stronger repulsive forces (Fig. 3g,h). Furthermore, the LDH membrane’s channel size (~ 3 Å) induces greater dehydration for Mg^2+^ ion. Through a combination of repulsive interactions from the positively charged surface and the relatively lower dehydration energy barrier for Li^+^ ion, the authors achieved Li^+^/Mg^2+^ selectivity.

Another approach to enhance Li^+^ ion selectivity is incorporating functional groups or molecules that preferentially bind to Li^+^ ion within the channel. For example, the selectivity filter of K^+^ ion channels in biological systems is lined with eight carbonyl oxygen atoms [68]. This configuration mimics the hydration coordination number of K^+^ ion (eight water molecules), replacing water molecules and providing a similar environment. These oxygen atoms are spaced similarly to water molecules in the hydrated state, offsetting the energy cost of fully dehydrating K^+^ ion and achieving high K^+^ ion selectivity. Bing et al. implanted lithiophilic oligoether (4EO) into densely aligned 2D Covalent Organic Framework nanosheets to achieve a Li^+^/Mg^2+^ selectivity of 12 in a single salt solution [33]. The 4EO contains four oxygen atoms, closely matching Li^+^ ion’s hydration coordination number (4–6 water molecules), allowing it to replace water molecules in the hydration shell. This coordination effect compensates for the dehydration energy cost of Li^+^ ion, enabling Li^+^ ion enrichment within the channel and resulting in high selectivity. Similar effects could be achieved by incorporating crown ethers into 2D channel membranes, as discussed in the following section.

### Li^+^/Mg^2+^ selectivity in diffusion step with 2D channel membrane

**Fig. 4:**
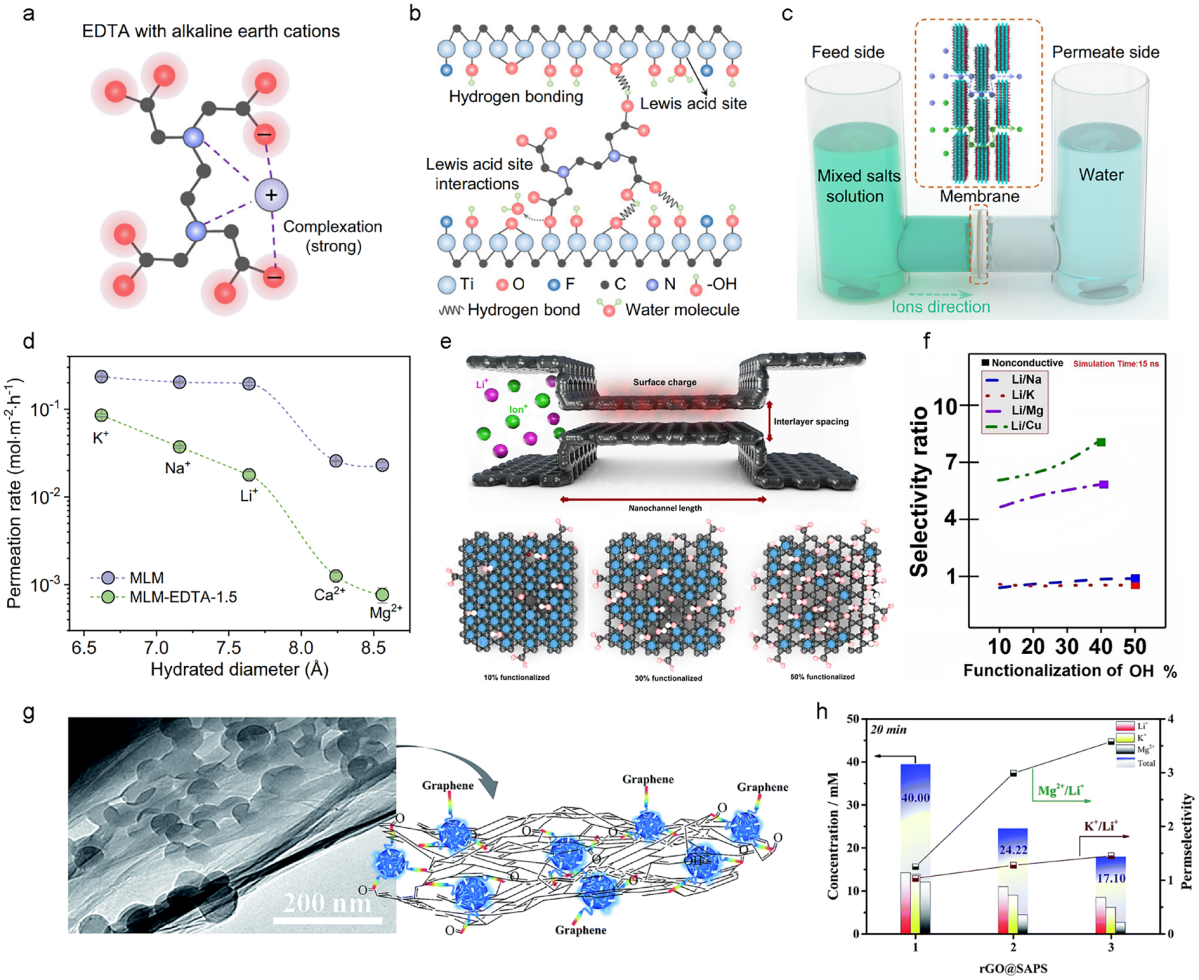
Li^+^/Mg^2+^ selectivity in diffusion step with 2D channel membrane. **a** Schematic of the interaction between ethylenediaminetetraacetic acid (EDTA) molecule and alkaline earth cations. **b** Schematic of cross-linking mechanism between Ti_3_C_2_T_*x*_ nanosheets and EDTA molecule. **c** Schematic of U-shaped device used for ion permeation measurements through Ti_3_C_2_T_*x*_-EDTA membrane. **d** Comparision of permeation rates of hydrated cations through Ti_3_C_2_T_*x*_ membrane and Ti_3_C_2_T_*x*_-EDTA membrane. **e** Schematic of three criteria, including interlayer spacing, surface charge, and channel length used in MD simulations (top). Schematic of the different interactions under the increasing percentage of functional groups (bottom). **f** Calculated selectivity ratio as a function of percentage of the hydroxyl groups. **g** Schematic of self-assembled multilayer reduced graphene oxide membrane with sulfonated amino-polystyrene nanosphere (rGO@SAPS). **h** Permselectivity and ion flux of rGO@SAPS with different mass ratios of SAPS. **a**–**d** Reproduced with permission [29] under the terms of the CC-BY Creative Commons Attribution 4.0 International License (http://creativecommons.org/licenses/by/4.0/). **e**,**f** Reproduced with permission [70]. Copyright 2020, Elsevier B.V. **g**, **h** Reproduced with permission [71]. Copyright 2018, The Royal Society of Chemistry

Due to its higher ion valence, Mg^2+^ ion interacts more strongly with negative charges, which can hinder its diffusion through negatively charged channels compared to Li^+^ ion. For example, Ethylenediaminetetraacetic acid (EDTA), a molecule containing several deprotonated carboxyl groups, binds more strongly to alkaline earth metal ions than to alkali metal ions due to the presence of negatively charged oxygen atoms and two electronegative nitrogen atoms (Fig. 4a). Xu et al. incorporated EDTA molecules into MXene (Ti_3_C_2_T_*x*_) membranes [29]. The carboxyl groups of EDTA were crosslinked to hydroxyl or oxygen groups on Ti_3_C_2_T_*x*_ through hydrogen bonding or Ti atoms through covalent bonding (Fig. 4b). This crosslinking prevents swelling in water, stabilizing the membrane for practical use. Permeation tests using a homemade U-shaped device demonstrated that strong interactions between EDTA and Mg^2+^ ion result in high selectivity for Li^+^ ion and other monovalent cations (Fig. 4c). The selectivity for Li^+^ ion and other monovalent cations increases at higher pH levels because more hydroxyl and carboxyl groups deprotonate, increasing the local charge density within the membrane, which further hinders Mg^2+^ ion diffusion.

However, excessive negative surface charge can reduce Li^+^/Mg^2+^ selectivity. Wang et al. used molecular dynamics simulations to observe Li^+^ ion and Mg^2+^ ion transport through carbon nanotubes (CNTs) with varying negative surface charges (0 to –10*e*, where *e* is the elementary charge) [69]. They found that while Li^+^/Mg^2+^ selectivity increases as the surface charge rises from 0 to –6*e*, further increases in surface charge result in slower Li^+^ ion transport, eliminating Li^+^ ion selectivity. Typically, stronger electrostatic interactions occur for cations with higher valence, so faster transport of Mg^2+^ ion than Li^+^ ion was unexpected. The authors attributed this to Li^+^ ion developing a more disordered hydration shell with stronger negative surface charges, causing Li^+^ ion to interact more strongly with the CNT walls. In contrast, Mg^2+^ ion retained a more ordered hydration shell, leading to weaker interactions with the CNT walls and faster transport. Therefore, optimal surface charge tuning is critical for enhancing Li^+^ ion selectivity.

Li^+^ ion selectivity can also vary based on the type of functional group that creates the negative surface charge within the channel. Graphene oxide layers feature epoxy, hydroxyl, and carboxyl groups, each interacting differently with ions as binding sites. A. Razmjou et al. conducted molecular dynamics simulations to observe the effects of functional group type, degree of functionalization, and channel size on ion transport through GO surfaces (Fig. 4e) [70]. They found that increasing hydroxyl group functionalization on the GO surface correlates with increased Li^+^/Mg^2+^ selectivity (Fig. 4f), which can be attributed to the increased negative surface charge hindering Mg^2+^ ion transport. Additionally, the authors analyzed Li^+^ ion selectivity for different functional groups (epoxy, hydroxyl, carboxyl, sulfonate) at a GO interlayer spacing of 9.5 Å. Although Li^+^/Mg^2+^ selectivity increased with the number of functional groups, the trend varied slightly depending on the functional group type, likely due to differences in the distribution of functional groups on the surface or edge of graphene and the length of the functional group itself. Further analysis is needed to fully understand these effects.

Alkali metal ions and alkaline earth metal ions typically exhibit different binding affinities for sulfonate groups. Sulfonate groups have a higher binding affinity for Mg^2+^ ion than for Li^+^ ion. Zhao et al. created a membrane by conjugating sulfonated amino-polystyrene nanospheres (SAPS) with reduced graphene oxide (rGO) (Fig. 4g) [71]. The amino groups on the nanospheres formed amide bonds with carboxyl groups on GO. To prevent the dispersion of GO in water, hydrothermal methods were used to reduce it to rGO@SAPS. The resulting membrane had an interlayer spacing of 3.8–5.5 Å, leading to high dehydration energy barriers for Mg^2+^ ion. Additionally, the sulfonate groups on the nanospheres further inhibited Mg^2+^ ion diffusion due to their strong binding affinity for Mg^2+^ ion. The authors varied the ratio of SAPS within the rGO membrane and found that as the SAPS content increased, the interlayer spacing decreased, leading to reduced permeability but higher Li^+^/Mg^2+^ selectivity (Fig. 4h). This was attributed to the higher proportion of sulfonate groups with increasing SAPS content.

## Origin of Li^+^/Na^+^ selectivity

Although Li^+^ and Na^+^ ions share the same charge, Li^+^ ion has a smaller bare ion size (Li^+^: 1.2 Å, Na^+^: 1.9 Å), which leads to a larger hydrated ion size (Li^+^: 7.64 Å, Na^+^: 7.16 Å) and higher hydration energy (Li^+^: –475 kJ/mol, Na^+^: –365 kJ/mol) [46, 58]. To achieve Li^+^ ion selectivity during the dehydration process as ions enter the membrane, complex interactions must be considered.

Channel size and surface charge play a crucial role in Li^+^ ion selectivity during the dehydration process [14]. However, specific conditions regarding channel size and surface charge are not yet fully defined, and much research has focused on Li^+^/Na^+^ selectivity during the dehydration and diffusion process.

Molecules that interact specifically with Li^+^ ion can serve as binding sites to enhance Li^+^ ion selectivity. These molecules can selectively attract Li^+^ ion or act as ion transport channels, selectively lowering the energy barrier during Li^+^ ion dehydration. This approach can endow channels that previously lacked Li^+^/Na^+^ selectivity with the ability to discriminate between the two ions.

Moreover, introducing binding sites within the channel that exhibit low binding energy with Li^+^ ions allows for rapid binding site-to-binding site movement with minimal energy. If these binding sites exhibit lower affinity for Na^+^ ion than for Li^+^ ion, Li^+^ ions will move faster through the channel compared to Na^+^ ions, improving Li^+^ ion selectivity.

### Li^+^/Na^+^ selectivity in dehydration step with 2D channel membrane

If no surface charge or binding sites exist within the channel, and the surface is neutral, Li^+^ ion selectivity can be enhanced if the channel size is smaller than the hydrated ion size of Li^+^ ion, thus promoting dehydration. However, achieving Li^+^ ion selectivity in the dehydration step may require more precise conditions related to channel size. For example, Esfandiar et al. fabricated slit channels with a size of ~ 6.6–6.7 Å and observed no Li^+^/Na^+^ selectivity, even in neutral channels [48]. Reducing the channel size further could potentially yield Li^+^ ion selectivity in neutral environments.

Zhang et al. utilized ZIF-8, a type of Metal–Organic Framework, to fabricate a membrane that demonstrated Li^+^/Na^+^ selectivity [72]. ZIF-8 features neutral channels with a small pore size of approximately 3.4 Å. The authors hypothesized that the Li^+^ ion selectivity could be attributed to (i) the effect of channel size and (ii) van der Waals interactions between the water molecules surrounding the ZIF-8 framework and those hydrating the cations. Based on this hypothesis, they conducted molecular dynamics (MD) simulations to calculate ion mobility by varying the strength of van der Waals interactions between the water molecules around the framework and those around the ions. While the calculated ion mobility varied by approximately 24% due to changes in these interactions, Li^+^/Na^+^ selectivity remained almost unchanged (~ 5%). Therefore, the authors concluded that the observed Li^+^/Na^+^ selectivity primarily resulted from partial dehydration due to the small channel size.

It is well known that surface charge within the channel can help compensate for the energy cost of ion dehydration through interactions with the ion. Wen et al. demonstrated this using ion track-etched polyethylene terephthalate membranes [34]. The channel size of the PET membrane was approximately 6 Å, and carboxyl groups were generated on the membrane surface during fabrication. These carboxyl groups deprotonated in water (pH 7) to form a negatively charged surface. The authors observed that as the solution’s pH was lowered from 6 to 3, the extent of carboxyl group deprotonation decreased, leading to reduced Li^+^ ion transport and the loss of Li^+^ ion selectivity. This would appear that the negative surface charge facilitated Li^+^ ion dehydration, reducing the loading time [60, 72]. However, the specific role of negative surface charge in Li^+^ ion selectivity during dehydration remains unclear.

**Fig. 5:**
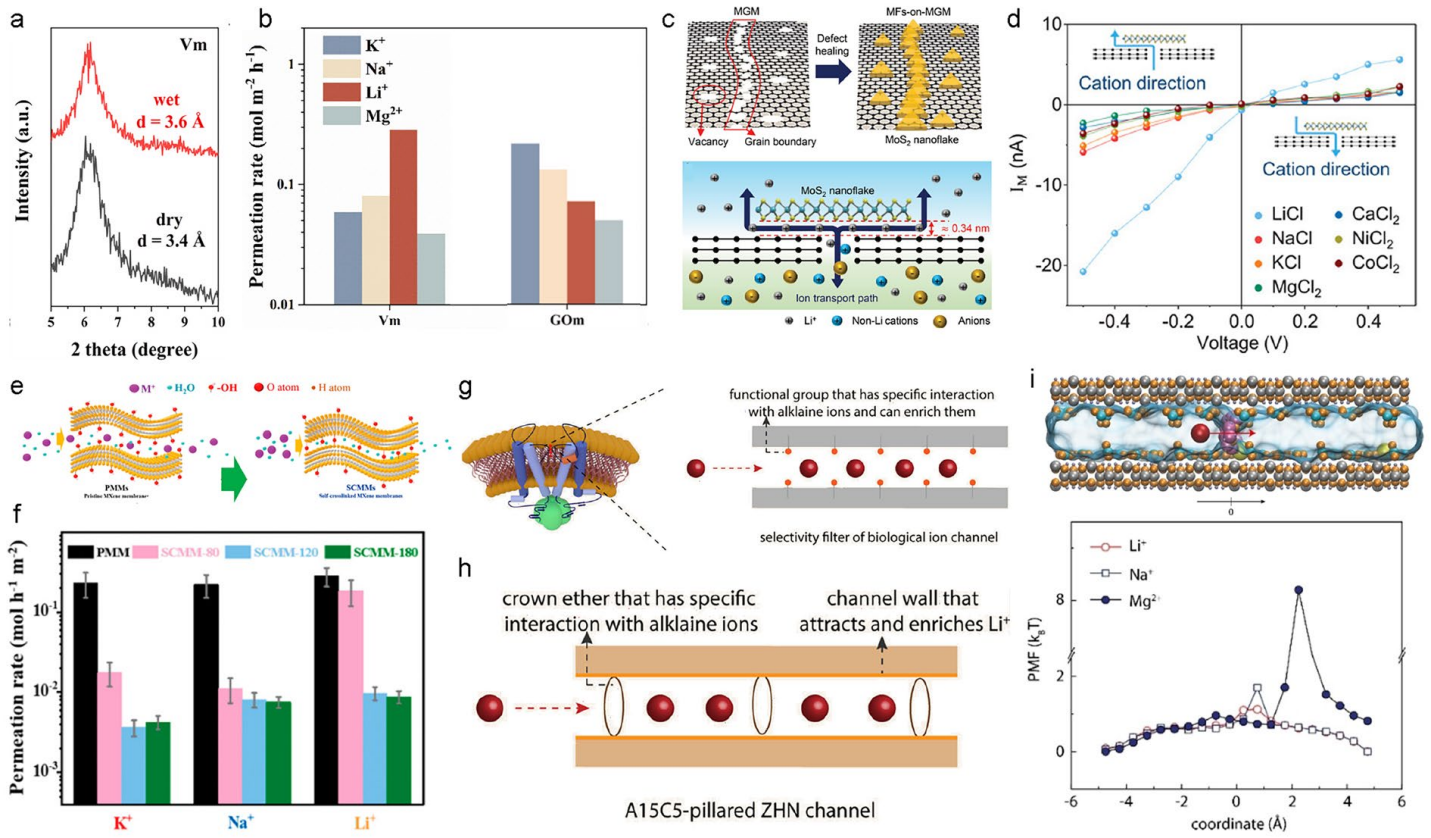
Li^+^/Na^+^ selectivity in the dehydration step with 2D channel membrane. **a** X-ray diffraction patterns for characterizing channel size of vermiculite membrane. **b** Comparison of the permeation rate between vermiculite and graphene oxide membrane. The channel size of vermiculite and graphene oxide membranes is 3.6 Å and 11.6 Å, respectively. **c**) Schematic of the fabricated MoS_2_ flakes on defective sites in the multilayer graphene membrane (MGM) and ion transport pathway. **d** *I–V* curve depends on cation transport direction. **e** Schematic of self-crosslinking process of Ti_3_C_2_T_*x*_ membrane. **f** Comparison of permeation rate with different self-crosslinking conditions of Ti_3_C_2_T_*x*_ membrane. **g** Schematic of specific interaction with alkali metal ions in the biological ion channel. **h** Schematic of fabricated crown-ether(A15C5)-pillared zinc hydroxide nitrate membrane to show similarity with biological ion channel. **i** Schematic of the simulation model (top). Potential of mean force (PMF) curves with simulation show a lower energy barrier for Li^+^ ion transport through an A15C5-pillared zinc hydroxide nitrate membrane (bottom). **a**–**b** Reprinted from [73]. Copyright 2023, Elsevier B.V. **c**-**d** Reproduced with permission [74]. Copyright 2023, Wiley–VCH GmbH. **e**, **f** Reproduced with permission [75]. Copyright 2019, American Chemical Society. **g**-**i** Reproduced with permission [80]. Copyright 2024, Wiley–VCH GmbH

Pang et al. fabricated a membrane using vermiculite, a type of clay mineral. The channel size, calculated by subtracting the thickness of the exfoliated vermiculite nanosheets (~ 1.1 nm) from the interlayer spacing, was approximately 3.6 Å (Fig. 5a) [73]. The vermiculite structure exhibited a partial substitution of Si^4+^ with Al^3+^, which generated a negative surface charge. X-ray Photoelectron Spectroscopy (XPS) further confirmed the presence of polar functional groups, likely arising from Si–O bonds, on the surface of the membrane. The fabricated vermiculite membrane demonstrated a Li^+^/Na^+^ selectivity value of 3.6. The small channel size of the vermiculite membrane likely facilitated the dehydration of both Li^+^ and Na^+^ ions, potentially enhancing the selectivity. The authors compared these results with Li^+^/Na^+^ selectivity measurements from water-swollen graphene oxide membranes (channel size ~ 11.5 Å) and concluded that the Li^+^ ion selectivity of the vermiculite membrane was primarily due to the dehydration effect from the small channel size (Fig. 5b). They also suggested that the size of the hydrated structure of dehydrated ions might further influence selectivity. However, the Li^+^/Na^+^ selectivity of the vermiculite membrane may be affected not only by the channel size but also by surface functional groups and surface charge, which requires additional analysis for a more comprehensive understanding.

Chang et al. fabricated MoS_2_ flakes-on-multilayer graphene membranes (MFs-on-MGM) using metal–organic chemical vapor deposition (MOCVD) to selectively grow MoS_2_ flakes on inborn defects in graphene layers, effectively healing the defects (Fig. 5c) [74]. The resulting membrane had a channel size of ~ 3.4 Å, smaller than the hydrated size of Li^+^ ion (7.64 Å). The authors confirmed that the membrane exhibited Li^+^/Na^+^ selectivity due to the small channel size. The selectivity may have been enhanced by negative surface charges at graphene defects and grain boundaries, where dangling bonds generated negative charge. These negative charges facilitated cation transport through the membrane, as evidenced by current measurements showing higher currents along the cation transport direction (Fig. 5d). The authors used finite-element analysis (FEA) to confirm that these negative charges led to non-symmetric current due to the facilitated movement of cations through graphene defects. Li^+^ ion selectivity was attributed to the differences in ion affinity at the membrane’s two ends, a phenomenon likely related to negative surface charges, though further investigation is needed to clarify the mechanism.

Lu et al. utilized self-crosslinking of hydroxyl groups on the surface of MXene (Ti_3_C_2_T_*x*_) to fabricate highly stable MXene membranes (Fig. 5e) [75]. By adjusting the temperature, the degree of hydroxyl group crosslinking was controlled, with Fourier transform infrared spectroscopy (FTIR) confirming a gradual decrease in hydroxyl groups as crosslinking increased. The channel sizes at the four temperature conditions tested (RT, 80 °C, 120 °C, 180 °C) were 6.6 Å, 5.6 Å, 5.5 Å, and 5.4 Å, respectively. As the channel size decreased, the permeation rate dropped significantly (Fig. 5f). However, even with small channel size differences of 0.1 Å, permeation rates varied depending on the ion, suggesting that factors beyond channel size, such as the energy barrier for dehydration, should be considered. It could be seen that the gradual reduction of hydroxyl groups influenced ion behavior, indicating a need for further analysis of factors affecting ion transport.

All of the 2D channel membranes discussed here feature channel sizes smaller than the hydrated size of Li^+^ ions, as well as negative surface charges. However, the exact role of surface charge in Li^+^/Na^+^ selectivity is often not well defined. Proper tuning of negative surface charge could selectively lower the energy barrier for partial dehydration of specific cations as they enter the membrane, thereby enhancing Li^+^/Na^+^ selectivity [53]. For example, Kim et al. used graphene oxide to facilitate fast Na^+^ ion transport and high cation selectivity for use in osmotic energy generation. The small channel size of GO (4.5 Å) and the negative surface charge from carboxyl groups reduced the dehydration energy barrier for Na^+^ ions, enabling faster transport compared to K^+^ ions [76]. However, selective lowering of the dehydration energy barrier for Li^+^ ion via surface charge tuning to achieve Li^+^/Na^+^ selectivity has not yet been reported.

In addition, the binding sites within 2D channel membranes can vary, playing a critical role in Li^+^ ion selectivity during the dehydration step. For example, binding sites with high affinity for Li^+^ ion can selectively allow Li^+^ ion to enter the membrane, enriching Li^+^ ion within the channel. These binding sites can be tuned by introducing specific functional groups or molecules into the membrane channels.

Crown ethers are cyclic molecules known for their specific interactions with alkali metal ions. Due to their specific interaction, crown ethers have been widely used to achieve alkali metal ion selectivity [77]. Biological ion channels achieve high selectivity for specific ions through a combination of angstrom-scale channel sizes and specific functional group arrangements (Fig. 5g) [78, 79]. Yu et al. applied this principle by intercalating 1-Aza-15-crown-5 (A15C5), a crown ether with specific interactions with Li^+^ ions, into layered zinc hydroxide nitrate (ZHN) (Fig. 5h) [80]. In addition to the specific interaction, A15C5 also causes a size exclusion effect due to its narrow pore size. A15C5 has a narrow pore size (~ 1.7 Å), allowing only small Li^+^ ion (1.2 Å bare ion size) to pass through. However, Na^+^ ion, with a bare ion size of 2 Å, requires a distortion of A15C5’s configuration to pass, leading to a higher energy requirement compared to Li^+^ ion, as confirmed by MD simulations using Potential of Mean Force (PMF) calculations (Fig. 5i). Based on this size exclusion effect and specific interaction, the A15C5-pillared ZHN membrane selectively allows Li^+^ ions to enter during the dehydration step, enriching Li^+^ ions within the channel.

### Li^+^/Na^+^ selectivity in diffusion step with 2D channel membrane

**Fig. 6:**
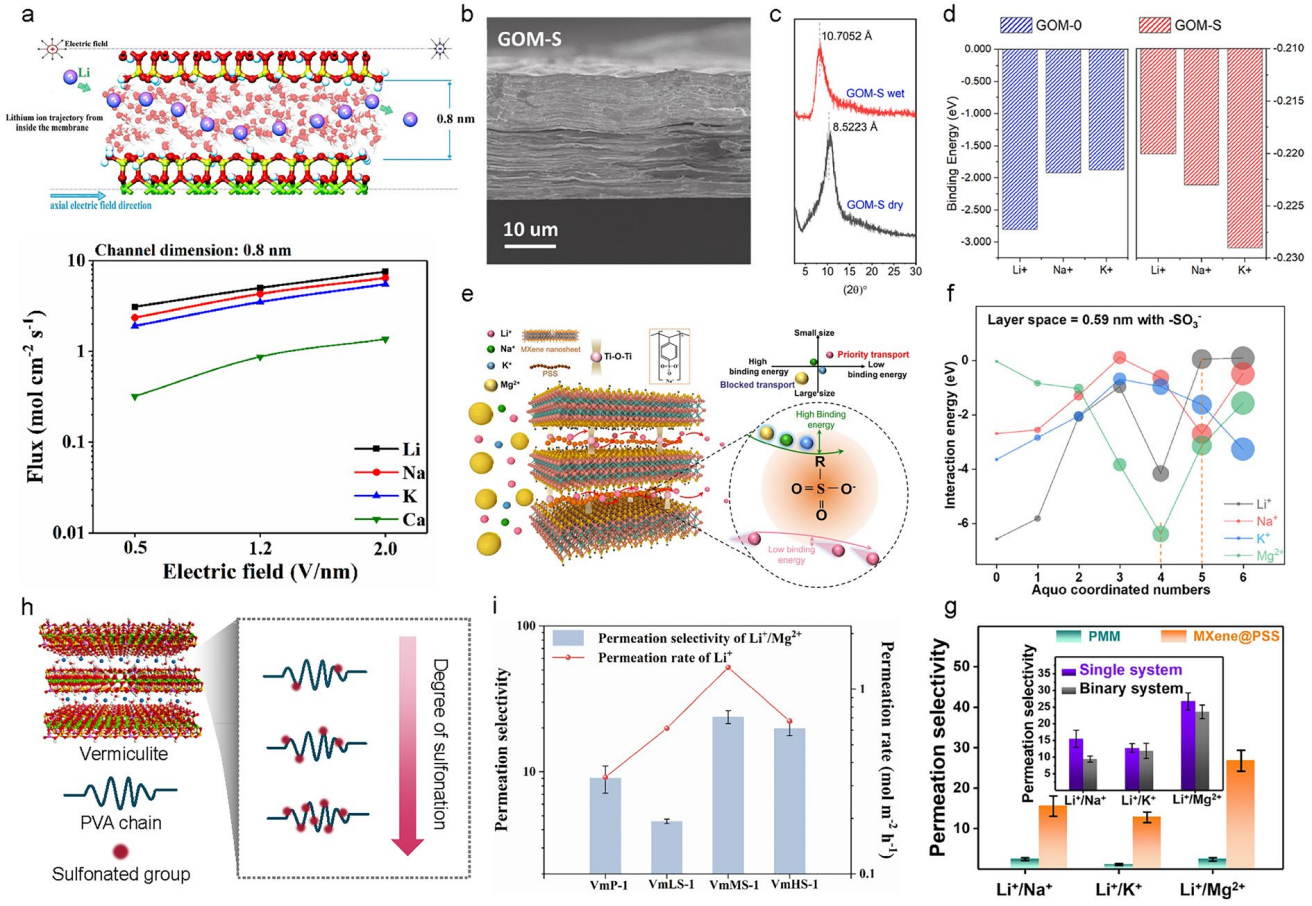
Li^+^/Na^+^ selectivity in diffusion step with 2D channel membrane. **a** Schematic of Li^+^ ion transport through vermiculite with a channel size of 0.8 nm (top). The ionic flux of vermiculite membrane with a channel size of 0.8 nm (bottom). **b** Scanning electron microscopy image of poly(sodium 4-styrene sulfonate) (PSS) molecules introduced graphene oxide membrane (GOM-S). **c** X-ray diffraction patterns of GOM-S for both dry and wet states. **d** Binding energy between alkali metal ions and the functional group on GOM and GOM-S based on density function theory (DFT) calculations. **e** Schematic of PSS introduced Ti_3_C_2_T_*x*_ membrane. **f** Interaction energy between sulfonate group and cations (Li^+^, Na^+^, K^+^, Mg^2+^ ions) with different hydration number. **g** Comparison of permeation selectivity between Ti_3_C_2_T_*x*_ membrane and PSS introduced Ti_3_C_2_T_*x*_ membrane. **h** Schematic of sulfonate polyvinyl alcohol (SPVA) with different sulfonation degrees introduced vermiculite membrane.** i**) Comparison of permeation selectivity with different sulfonation degrees of SPVA-introduced vermiculite membranes. **a** Reproduced from permission [81]. Copyright 2019, Elsevier Ltd. **b**–**d** Reproduced from permission [39]. Copyright 2023, Elsevier B.V. **e**–**g**) Reproduced from permission [82]. Copyright 2021, Wiley‐VCH GmbH. **h**,**i** Reproduced from permission [73]. Copyright 2023, Elsevier B.V

When the channel size is larger than the hydrated ion size of Li^+^ ion, surface charge plays a crucial role in Li^+^/Na^+^ selectivity during diffusion. Surface charge can induce partial dehydration of hydrated ions, leading to a more compact hydration shell for Li^+^ ions, which enhances their mobility within the channel. A. Razmjou et al. conducted MD simulations to observe ion diffusion through 2D channels [81]. Vermiculite, with its layered structure, exhibited surface charge. The authors calculated diffusion coefficients for Li^+^, Na^+^, K^+^, and Ca^2+^ ions in channel sizes of 0.4 nm and 0.8 nm. In both cases, Li^+^ ions exhibited faster diffusion than Na + ions (Fig. 6a). This was attributed to the partial dehydration of Li^+^ ions due to the surface charge, resulting in a compact hydration shell and higher ion mobility [34].

Sulfonate groups, with their negative charge, can accelerate ion transport and promote Li^+^/Na^+^ selectivity during diffusion without requiring dehydration. Liu et al. intercalated poly(sodium 4-styrene sulfonate) (PSS) as a spacing agent into graphene oxide membranes [39]. Scanning Electron Microscope (SEM) images confirmed that the layered structure of the GO membrane remained intact after PSS intercalation (Fig. 6b). Normally, GO experiences significant swelling in water due to water adsorption into the interlayer space. However, PSS acted as a cross-linker, preventing significant swelling, as confirmed by X-ray diffraction (XRD) measurements, which indicated a channel size of ~ 10.7 Å (Fig. 6c). Despite the large channel size relative to the hydrated size of Li^+^ ion, Li^+^/Na^+^ selectivity was observed, attributed to the interaction between sulfonate groups and alkali metal ions (Fig. 6d).

The interaction between sulfonate groups and ions can be enhanced following dehydration, potentially yielding higher Li^+^/Na^+^ selectivity. Lu et al. incorporated PSS, a chain polymer containing sulfonate groups, into 2D MXene (Ti_3_C_2_T_*x*_) membranes (Fig. 6e) [82]. The MXene/PSS composite membrane was produced by a simple filtration method, mixing MXene nanosheet and PSS dispersions. The fabricated membrane had a channel size of 5.9 Å, small enough to induce dehydration of both Li^+^ and Na^+^ ions. MD simulations were used to calculate the stable hydration number of dehydrated ions within the membrane. The results showed that the stable hydration number varied with ion species, affecting their interaction with sulfonate groups in the membrane (Fig. 6f). This MXene/PSS composite membrane achieved a high Li^+^/Na^+^ selectivity of 15.5 in a single salt solution (Fig. 6g).

However, excessive introduction of sulfonate groups can reduce Li^+^ ion selectivity. Pang et al. introduced sulfonated polyvinyl alcohol (SPVA) into 2D vermiculite membranes, achieving higher Li^+^/Na^+^ selectivity (3.6 for pristine vermiculite and 14.1 for SPVA-modified vermiculite) [73]. To further analyze the effect of sulfonate groups on selectivity, they varied the proportion of sulfonate groups in SPVA (Fig. 6h). As the sulfonate group content increased, Li^+^ ion transport increased, but beyond a certain threshold, both Li^+^ ion mobility and selectivity decreased (Fig. 6i). This decline was attributed to counterion condensation at the binding sites, preventing ion transport [83]. Therefore, precise control of binding site proportions may be necessary to maximize Li^+^/Na^+^ selectivity.

### Challenges and outlook

With the increasing demand for lithium, various 2D channel membranes have been investigated for their potential in extracting Li^+^ ions from aqua-based resources. To ensure the successful application of membrane technologies for aqua-based lithium extraction, it is crucial to enhance Li^+^ ion selectivity, particularly in seawater, where lithium exists in large absolute amounts. While many studies have reported promising Li^+^/Mg^2+^ selectivity, Li^+^ ion selectivity over Na^+^ ions, which are abundant in seawater, remains relatively low due to the similar properties of monovalent cations. Achieving high Li^+^/Na^+^ selectivity requires a more nuanced approach, carefully considering the rules and principles governing each step of ion permeation through membranes.

Biological ion channels have been extensively studied due to their high selectivity between monovalent cations. It is widely understood that this high selectivity is primarily achieved during the dehydration process [51, 68]. For example, in K^+^ ion channels, the selectivity filter attracts K^+^ ions through strong interactions involving the number and arrangement of carbonyl groups and pore size. However, these strong interactions can create significant transport hindrances during the diffusion process, reducing selectivity for the target ion [53, 57]. The short channel length of K^+^ ion channels (approximately 12 Å) minimizes transport hindrance, allowing the ion permeation process to experience fewer counteracting effects between dehydration and diffusion [53, 84].

In contrast, 2D channel membranes, constructed by stacking nanosheets, have comparatively longer channel lengths due to the lateral size of the nanosheets (up to the micrometer scale). This increased length can exacerbate the counteracting effects of dehydration and diffusion. Therefore, achieving high Li^+^ ion selectivity in 2D channel membranes requires careful consideration of the energy barriers in both the dehydration and diffusion steps to manage these counteracting effects appropriately.

To finely control these counteracting effects, clear rules and principles are needed to understand how key variables influence each step. This review has examined various studies that demonstrate Li^+^ ion selectivity using 2D channel membranes. Many studies show promising results by manipulating variables associated with one of the two permeation steps. However, there is a lack of research that comprehensively addresses how channel size, surface charge, and binding sites affect the counteracting effects across both the dehydration and diffusion steps. This limitation points to the need for better-defined rules and principles governing how key factors influence ion transport, which could be a major barrier to achieving high Li^+^/Na^+^ selectivity in 2D channel membranes.

Despite these challenges, 2D channel membranes remain highly attractive due to their chemical stability, tunability, and ease of fabrication and scaling up [85–87]. By developing a clear understanding of the factors affecting ion transport and applying this knowledge to design membranes that control both dehydration and diffusion, 2D channel membranes could become an ideal membrane technology for lithium-ion extraction.

## Data Availability

The datasets used and/or analysed during the current study are available from the corresponding author on reasonable request.
